# The Treatment of Cough in Phthisis

**Published:** 1892-06-04

**Authors:** 


### The Practitioner's Mirror and Retrospect.

*The Editor will be glad to receive offers of co-operation and contributions from members of the profession. All letters should be addressed to* The Editor, The Lodge, Porchester Square, London, W.]

### MEDICINE.

### THE TREATMENT OF COUGH IN PHTHISIS.

There are few subjects in our daily work of greater importance than this, both from its effects on the individual patient and from the numbers who are concerned. No specific against the bacillus as yet has prevailed. One-seventh of the human race are destroyed by him. Neither Koch, nor the apostles of cantharidine, or goats' serum have made much impression on his invading hosts, though we learn from post-mortem evidence that a very large proportion of those who are attacked by him recover completely. Whether we aim at such recovery in the early stages, or merely strive to postpone the fatal event, one of the greatest difficulties which we encounter is the proper treatment of the harassing exhausting cough which pulls down our patient's strength as fast as we build it up. Now this cough is produced in many various ways in the course of the disease. Irritation in any part of the respiratory tract, and even elsewhere as in the ear or bowels will bring it on while the lungs are in an abnormal state, and no routine treatment short of rendering the patient insensible will succeed in all cases. The essential point in a confessedly difficult subject is to diagnose and appropriately treat the particular form of irritation. This patient is racked with cough every evening till you find that there is hyperexia at a certain hour, which is relieved by an anti-pyretic. Another coughs from slight implication of the pleura relieved at once by iodine liniment or a poultice. Another has ulcers in the larynx, or even in the pharynx itself, while a relaxed uvula, or fresh bronchial cold may cause it, over and over again, in other patients. How far can we dispense with opiates and the disturbance of the digestion which they so often produce? Dr. Mitchell Bruce has recently described many of the varieties of phthisical cough and their treatment from a clinical, rather than a pathological, point of view. First of all he notes that there is often a wearisome cough in the evening felt by patients who think they are well enough to remain up in the family circle enjoying the conversation of friends. For these he insists on an early bed-time, regarding the cough as an indication of fatigue. It would seem, indeed, to be at times only a mark of the evening hectic, relieved in some cases by a full dose of quinine or antipyrin two hours before the climax, but it is always an indication for rest and careful treatment. Other patients suffer from violent cough at bedtime while undressing. He rightly lays stress on a warmed bed and bedroom, and the importance of undressing by degrees without increasing the rate of respiration. If necessary, he gives a little simple syrup, or one with a dose of morphine to be held in the mouth for some time before it is swallowed, so as to moisten the whole pharynx. A cough during the night is often best relieved by a little food kept warm over a night-light, to which may be added, in obstinate cases, some stimulant, or even a morphine preparation. Patients, especially those who retire early, are plagued by cough in the early morning, when the lungs endeavour to free themselves of the accumulations of the night. The rational plan is to aid them in this by an early meal, instead of hindering them by giving a sedative. Morphine and syrups must now be eschewed, and some hot food administered as soon as they wake. During the day we find a violent attack of coughing often follows a meal, especially if there is any dyspepsia. Difficult as this is to treat, careful diet and complete rest immediately after taking food, will often give relief. A distended stomach pressing on the lung where a cavity exists with thickened walls, may set up violent paroxysms of cough, but a blister over the cavity or repeated painting with iodine over the same region often gives ease without the use of sedatives. Wherever the cough is due to local irritation of the pleura or of the surface of the lung, and not to congestion or obstruction of the bronchioles with mucus, local counter-irritants, bandages, and strapping should entirely or largely replace sedatives. Many consumptives have a succession of flying pains in different places, with a hacking cough, which can thus be relieved without any internal treatment whatever. An alkaline draught to correct acidity in the intervals of digestion will sometimes stop a cough, for, as Brunton points out, though the stomach itself does not cause cough, disturbance in it may reflexly excite parts of the lung which are in a state of chronic irritation from disease. Hence, too, the importance of regulating the bowels, and sometimes of bracing up the heart and vessels by digitalis and other tonics.

In short, to treat rationally, we must consider every part of the machine, and rely as little as possible on syrups and sedatives. Expectorants are of use when the secretion is thick and profuse, but if it comes from a cavity a local counter-irritant is often of more value. Again, when we have succeeded in rendering the expectoration easy, we may endeavour to diminish its quantity by a combination of atropine and morphine, which lessen the amount and allay the irritability of the centre for coughing. One great value of the turpentine and creosote preparations, which so many patients find benefit from, undoubtedly arises from the diminished secretion and irritability of the mucous surfaces, which these substances cause without rendering the sputa thick and tough. Other remedies for excessive cough are ice held in the mouth, sprays containing menthol, warm water, or alum solutions, poultices on the chest, and compresses over the trachea, combined with rest and the avoidance of cold or impure air. The pharynx easily becomes irritable unless we protect it by astringent and antiseptic gargles from the effects of the constant passage of tuberculous matter, and this leads us to the question of laryngeal troubles. Apart from the question of Koch's treatment, which will probably be rendered ere long far more perfect, we must rely chiefly on local treatment by astringents and sedatives, such as inhalations of benzoin, sprays of cocaine, and insufflation of morphine and starch. A spray containing lactic acid and morphine is one of the most serviceable. Where ulceration exists, Heryng's method of rubbing in a 20 or 50 per cent. solution of lactic acid by cotton wool on a probe is invaluable. Cocaine should be applied before and after the operation, and, if necessary, the diseased parts may be scraped with a curette. Frequent soothing inhalations, compresses, and counter-irritants on the outside of the neck, combined with this method of treatment often effect a wonderful improvement, but in the worst cases feeding through the soft tube or tracheotomy must be resorted to. In conclusion, the first thing to be done for the relief of a phthisical cough is to determine how it is caused, and then we shall find that we can ordinarily use a local remedy in preference to a general sedative.

